# {2,2′-[5-Bromo­pyridine-2,3-diylbis(nitrilo­methyl­idyne)]diphenolato}chlorido(dimethyl­formamide)manganese(III)

**DOI:** 10.1107/S1600536809049484

**Published:** 2009-11-25

**Authors:** Hai Xie, Shuangming Meng, Yongjun Zhu, Peiwan Bai

**Affiliations:** a College of Chemistry & Chemical Engineering, Shanxi Datong University, Shanxi 037009, People’s Republic of China

## Abstract

In the title complex, [Mn(C_19_H_12_BrN_3_O_2_)Cl(C_3_H_7_NO)], the Mn^III^ ion is coordinated by two N and two O atoms from the tetra­dentate Schiff base ligand, one O atom from the dimethyl­formamide ligand and a Cl anion in a distorted octa­hedral geometry. In the crystal structure, weak inter­molecular C—H⋯Cl hydrogen bonds link the mol­ecules into centrosymmetric dimers with a short distance of 3.878 (3) Å between the centroids of the aromatic rings.

## Related literature

For related structures, see: Li *et al.* (2008 ▶); Eltayeb *et al.* (2008*a* ▶,*b* ▶); Fei & Fang (2008 ▶).
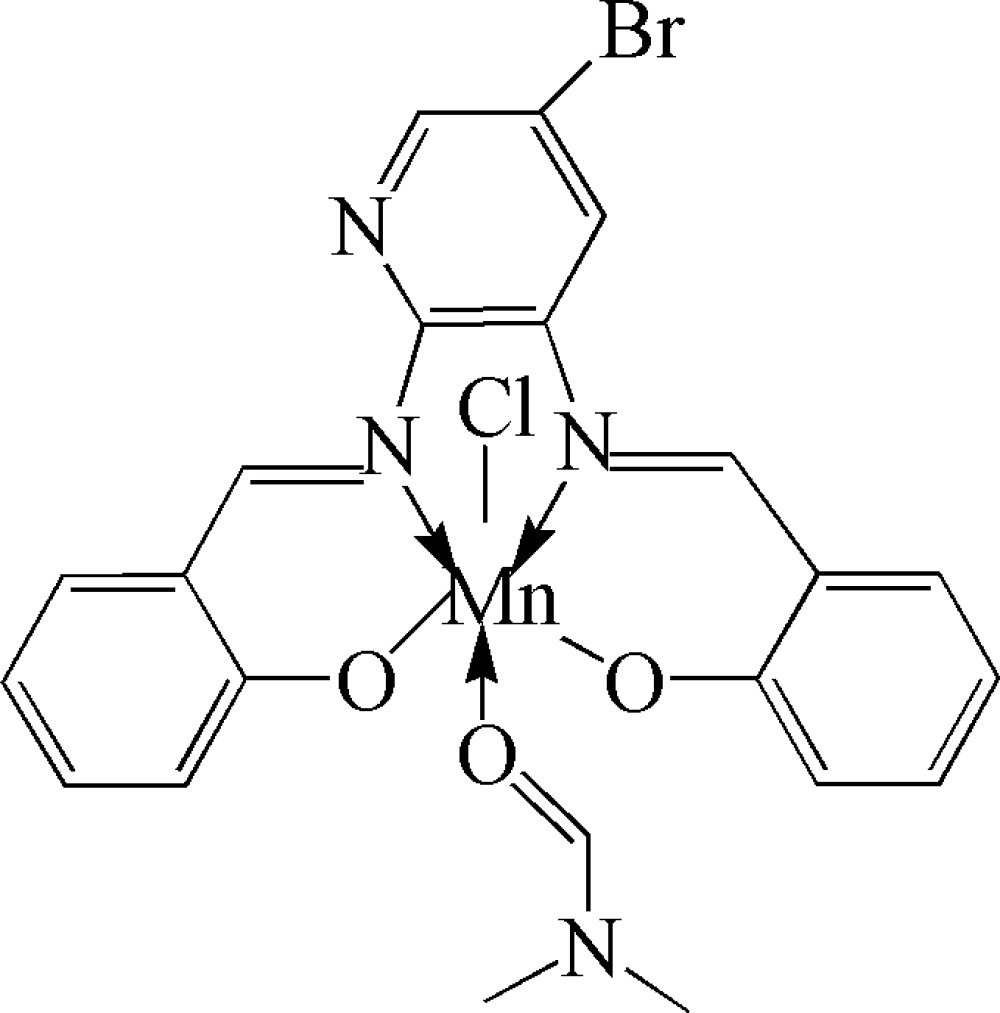


## Experimental

### 

#### Crystal data

[Mn(C_19_H_12_BrN_3_O_2_)Cl(C_3_H_7_NO)]
*M* *_r_* = 557.71
Monoclinic, 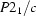

*a* = 13.2834 (11) Å
*b* = 15.4971 (13) Å
*c* = 12.2314 (11) Å
β = 117.143 (1)°
*V* = 2240.6 (3) Å^3^
*Z* = 4
Mo *K*α radiation
μ = 2.52 mm^−1^
*T* = 293 K
0.31 × 0.21 × 0.19 mm

#### Data collection

Bruker APEXII CCD area-detector diffractometer
Absorption correction: multi-scan (*SADABS*; Sheldrick, 2008*a* ▶) *T*_min_ = 0.508, *T*_max_ = 0.646
10906 measured reflections
3945 independent reflections
3238 reflections with *I* > 2σ(*I*)
*R*_int_ = 0.022

#### Refinement

*R*[*F* ^2^ > 2σ(*F* ^2^)] = 0.039
*wR*(*F* ^2^) = 0.116
*S* = 1.06
3945 reflections
291 parameters
H-atom parameters constrained
Δρ_max_ = 1.30 e Å^−3^
Δρ_min_ = −0.52 e Å^−3^

### 

Data collection: *APEX2* (Bruker, 2004 ▶); cell refinement: *SAINT-Plus* (Bruker, 2001 ▶); data reduction: *SAINT-Plus*; program(s) used to solve structure: *SHELXS97* (Sheldrick, 2008*b* ▶); program(s) used to refine structure: *SHELXL97* (Sheldrick, 2008*b* ▶); molecular graphics: *SHELXTL* (Sheldrick, 2008*b* ▶); software used to prepare material for publication: *SHELXTL*.

## Supplementary Material

Crystal structure: contains datablocks I, global. DOI: 10.1107/S1600536809049484/cv2658sup1.cif

Structure factors: contains datablocks I. DOI: 10.1107/S1600536809049484/cv2658Isup2.hkl

Additional supplementary materials:  crystallographic information; 3D view; checkCIF report

## Figures and Tables

**Table 1:** Hydrogen-bond geometry (Å, °)

*D*—H⋯*A*	*D*—H	H⋯*A*	*D*⋯*A*	*D*—H⋯*A*
C19—H19⋯Cl1^i^	0.93	2.81	3.691 (2)	159

Symmetry code: (i) 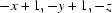.

**Fig. 1.**
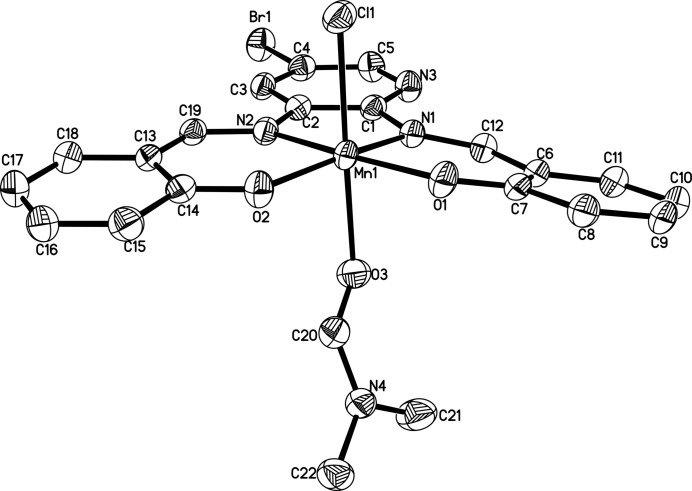
The molecular structure of the title compound with the atom-labelling scheme. Displacement ellipsoids are drawn at the 30% probability level. All H-atoms are omitted for clarity.

## supplementary crystallographic information

### Comment

Because of their interesting structures and wide potential applications, the synthesis and structural investigation of Schiff base complexes have been given much attention. Furthermore, these types of complexes play an important part in the development of coordination chemistry as well as inorganic biochemistry, catalysis, optical materials and so on (Li *et al.*, 2008; Fei & Fang, 2008).

The crystal structure of the title compound is shown in Fig. 1. The coordination sphere of the Mn^III^ ion is a slightly distorted octahedron, in which the four equatorial positions are occupied by two N atoms and two O atoms coming from the tetradentate Schiff base ligand, and the two axial ones with a *trans* conformation are occupied by one Cl ion and one O atom of the coordinated dimethylamine-methoxyl, respectively. The Mn—N, Mn—O and Mn—Cl bond lengths are basically consistant with those corresponding distances in other Mn-Schiff base complexes (Li *et al.*, 2008; Eltayeb *et al.*, 2008a, b). It is worth noting that centrosymmetric dimers with the short distance of 3.878 (3) Å between the centroids of aromatic rings are formed under the help of the weak intermolecular C—H···Cl hydrogen bond interaction (Table 1).

### Experimental

The Schiff base ligand was synthesized by condensation of 5-bromo-2,3-diaminopyridine and 2-hydroxy-benzaldehyde with the ratio 1:2 in ethanol. The synthesis of the title complex was carried out by reacting Mn(ClO_4_)_2_.6H_2_O, and the schiff-base ligand (1:1, molar ratio) in methanol. After the stirring process was continued for about one hour at room temperature, the mixture was filtered. The insoluble dark-brown solid was filtered out, dissolved in DMF and layered with ether. After one month, the block dark-brown crystals suitable for X-ray diffraction were obtained with a yield about 50%.

### Refinement

H atoms were placed in calcluated positions (C—H 0.93-0.96 Å), and were refined as riding atoms, with *U*_iso_(H) = 1.2-1.5*U*_eq_(C).

### Crystal data

**Table:**

[Mn(C_19_H_12_BrN_3_O_2_)Cl(C_3_H_7_NO)]	*F*(000) = 1120
*M**_r_* = 557.71	*D*_x_ = 1.653 Mg m^−^^3^
Monoclinic, *P*2_1_/*c*	Mo *K*α radiation, λ = 0.71073 Å
Hall symbol: -P 2ybc	Cell parameters from 2012 reflections
*a* = 13.2834 (11) Å	θ = 2.1–26.7°
*b* = 15.4971 (13) Å	µ = 2.52 mm^−^^1^
*c* = 12.2314 (11) Å	*T* = 293 K
β = 117.143 (1)°	Block, dark-brown
*V* = 2240.6 (3) Å^3^	0.31 × 0.21 × 0.19 mm
*Z* = 4	

### Data collection

**Table:**

Bruker APEXII CCD area-detector diffractometer	3945 independent reflections
Radiation source: fine-focus sealed tube	3238 reflections with *I* > 2σ(*I*)
graphite	*R*_int_ = 0.022
φ and ω scans	θ_max_ = 25.0°, θ_min_ = 2.2°
Absorption correction: multi-scan (*SADABS*; Sheldrick, 2008a)	*h* = −15→15
*T*_min_ = 0.508, *T*_max_ = 0.646	*k* = −13→18
10906 measured reflections	*l* = −14→13

### Refinement

**Table:**

Refinement on *F*^2^	Primary atom site location: structure-invariant direct methods
Least-squares matrix: full	Secondary atom site location: difference Fourier map
*R*[*F*^2^ > 2σ(*F*^2^)] = 0.039	Hydrogen site location: inferred from neighbouring sites
*wR*(*F*^2^) = 0.116	H-atom parameters constrained
*S* = 1.06	*w* = 1/[σ^2^(*F*_o_^2^) + (0.0643*P*)^2^ + 1.7733*P*] where *P* = (*F*_o_^2^ + 2*F*_c_^2^)/3
3945 reflections	(Δ/σ)_max_ = 0.001
291 parameters	Δρ_max_ = 1.30 e Å^−^^3^
0 restraints	Δρ_min_ = −0.52 e Å^−^^3^

### Special details

**Table:**

Geometry. All e.s.d.'s (except the e.s.d. in the dihedral angle between two l.s. planes) are estimated using the full covariance matrix. The cell e.s.d.'s are taken into account individually in the estimation of e.s.d.'s in distances, angles and torsion angles; correlations between e.s.d.'s in cell parameters are only used when they are defined by crystal symmetry. An approximate (isotropic) treatment of cell e.s.d.'s is used for estimating e.s.d.'s involving l.s. planes.
Refinement. Refinement of *F*^2^ against ALL reflections. The weighted *R*-factor *wR* and goodness of fit *S* are based on *F*^2^, conventional *R*-factors *R* are based on *F*, with *F* set to zero for negative *F*^2^. The threshold expression of *F*^2^ > σ(*F*^2^) is used only for calculating *R*-factors(gt) *etc*. and is not relevant to the choice of reflections for refinement. *R*-factors based on *F*^2^ are statistically about twice as large as those based on *F*, and *R*- factors based on ALL data will be even larger.

### Fractional atomic coordinates and isotropic or equivalent isotropic displacement parameters (Å^2^)

**Table:**

	*x*	*y*	*z*	*U*_iso_*/*U*_eq_	
Br1	0.45611 (4)	0.63147 (3)	0.36373 (4)	0.06441 (17)	
Mn1	0.81172 (4)	0.45734 (3)	0.12690 (5)	0.03870 (16)	
Cl1	0.68097 (8)	0.34011 (7)	0.05359 (10)	0.0587 (3)	
O1	0.9460 (2)	0.38529 (17)	0.1822 (2)	0.0521 (6)	
O2	0.7824 (2)	0.49890 (18)	−0.0267 (2)	0.0518 (6)	
O3	0.9317 (2)	0.56444 (18)	0.2006 (3)	0.0567 (7)	
N1	0.8278 (2)	0.45131 (18)	0.3010 (3)	0.0423 (7)	
N2	0.6755 (2)	0.54058 (18)	0.1157 (3)	0.0418 (7)	
N3	0.7332 (3)	0.4802 (2)	0.4180 (3)	0.0516 (8)	
N4	1.0806 (3)	0.6366 (2)	0.1995 (3)	0.0503 (8)	
C1	0.7383 (3)	0.4907 (2)	0.3157 (3)	0.0418 (8)	
C2	0.6585 (3)	0.5396 (2)	0.2180 (3)	0.0401 (8)	
C3	0.5713 (3)	0.5821 (2)	0.2312 (3)	0.0459 (8)	
H3	0.5175	0.6151	0.1681	0.055*	
C4	0.5694 (3)	0.5727 (2)	0.3378 (4)	0.0482 (9)	
C5	0.6486 (3)	0.5208 (3)	0.4285 (4)	0.0530 (9)	
H5	0.6428	0.5135	0.5009	0.064*	
C6	1.0097 (3)	0.3730 (2)	0.3939 (4)	0.0455 (9)	
C7	1.0197 (3)	0.3568 (2)	0.2899 (4)	0.0455 (9)	
C8	1.1146 (3)	0.3060 (3)	0.3007 (4)	0.0550 (10)	
H8	1.1228	0.2951	0.2304	0.066*	
C9	1.1925 (3)	0.2733 (3)	0.4095 (5)	0.0618 (11)	
H9	1.2519	0.2399	0.4125	0.074*	
C10	1.1839 (3)	0.2892 (3)	0.5137 (4)	0.0623 (11)	
H10	1.2372	0.2675	0.5888	0.075*	
C11	1.0937 (3)	0.3385 (3)	0.5056 (4)	0.0559 (10)	
H11	1.0881	0.3496	0.5774	0.067*	
C12	0.9159 (3)	0.4184 (2)	0.3953 (3)	0.0463 (8)	
H12	0.9177	0.4252	0.4717	0.056*	
C13	0.6144 (3)	0.5898 (2)	−0.0885 (3)	0.0432 (8)	
C14	0.7045 (3)	0.5503 (2)	−0.1064 (3)	0.0450 (8)	
C15	0.7093 (4)	0.5706 (3)	−0.2114 (4)	0.0555 (10)	
H15	0.7676	0.5487	−0.2254	0.067*	
C16	0.6292 (4)	0.6232 (3)	−0.2976 (4)	0.0620 (11)	
H16	0.6349	0.6356	−0.3689	0.074*	
C17	0.5388 (4)	0.6590 (3)	−0.2831 (4)	0.0605 (11)	
H17	0.4853	0.6937	−0.3438	0.073*	
C18	0.5322 (3)	0.6420 (3)	−0.1807 (4)	0.0528 (9)	
H18	0.4728	0.6645	−0.1693	0.063*	
C19	0.6052 (3)	0.5823 (2)	0.0182 (3)	0.0418 (8)	
H19	0.5438	0.6091	0.0213	0.050*	
C20	0.9824 (3)	0.5958 (3)	0.1477 (4)	0.0518 (9)	
H20	0.9484	0.5902	0.0627	0.062*	
C21	1.1408 (4)	0.6469 (4)	0.3284 (4)	0.0799 (14)	
H21A	1.1098	0.6946	0.3532	0.120*	
H21B	1.2191	0.6579	0.3515	0.120*	
H21C	1.1344	0.5952	0.3680	0.120*	
C22	1.1358 (4)	0.6716 (3)	0.1327 (4)	0.0620 (11)	
H22A	1.0857	0.6687	0.0462	0.093*	
H22B	1.2030	0.6390	0.1508	0.093*	
H22C	1.1558	0.7307	0.1562	0.093*	

### Atomic displacement parameters (Å^2^)

**Table:**

	*U*^11^	*U*^22^	*U*^33^	*U*^12^	*U*^13^	*U*^23^
Br1	0.0571 (3)	0.0792 (3)	0.0650 (3)	0.0171 (2)	0.0349 (2)	0.0026 (2)
Mn1	0.0317 (3)	0.0426 (3)	0.0388 (3)	0.0076 (2)	0.0134 (2)	0.0029 (2)
Cl1	0.0422 (5)	0.0516 (5)	0.0713 (7)	0.0020 (4)	0.0165 (5)	−0.0049 (5)
O1	0.0397 (14)	0.0642 (17)	0.0499 (15)	0.0126 (12)	0.0184 (12)	0.0036 (12)
O2	0.0499 (15)	0.0583 (16)	0.0463 (14)	0.0128 (13)	0.0212 (12)	0.0064 (12)
O3	0.0516 (15)	0.0600 (17)	0.0592 (17)	−0.0085 (13)	0.0259 (14)	−0.0013 (13)
N1	0.0373 (15)	0.0417 (16)	0.0448 (16)	0.0038 (12)	0.0161 (13)	0.0031 (13)
N2	0.0378 (15)	0.0421 (16)	0.0402 (16)	0.0030 (12)	0.0133 (13)	0.0013 (12)
N3	0.0526 (19)	0.061 (2)	0.0447 (18)	0.0090 (16)	0.0252 (15)	0.0067 (14)
N4	0.0459 (18)	0.0497 (18)	0.057 (2)	0.0010 (14)	0.0248 (16)	0.0029 (14)
C1	0.0361 (18)	0.0406 (19)	0.047 (2)	0.0007 (15)	0.0179 (16)	−0.0015 (15)
C2	0.0362 (18)	0.0412 (18)	0.0413 (19)	−0.0008 (14)	0.0163 (15)	−0.0027 (14)
C3	0.0387 (19)	0.045 (2)	0.047 (2)	0.0038 (16)	0.0134 (16)	0.0002 (16)
C4	0.042 (2)	0.051 (2)	0.055 (2)	0.0018 (16)	0.0245 (18)	−0.0021 (17)
C5	0.057 (2)	0.057 (2)	0.050 (2)	0.0087 (19)	0.0292 (19)	0.0052 (18)
C6	0.0330 (18)	0.045 (2)	0.052 (2)	0.0016 (14)	0.0131 (16)	0.0069 (16)
C7	0.0298 (17)	0.0419 (19)	0.058 (2)	0.0004 (14)	0.0142 (17)	0.0061 (16)
C8	0.044 (2)	0.049 (2)	0.073 (3)	0.0061 (17)	0.028 (2)	0.0050 (19)
C9	0.033 (2)	0.054 (2)	0.089 (3)	0.0102 (17)	0.020 (2)	0.014 (2)
C10	0.039 (2)	0.060 (3)	0.070 (3)	0.0065 (18)	0.009 (2)	0.019 (2)
C11	0.043 (2)	0.059 (2)	0.053 (2)	−0.0006 (18)	0.0110 (18)	0.0064 (19)
C12	0.042 (2)	0.049 (2)	0.044 (2)	0.0027 (16)	0.0164 (17)	0.0019 (16)
C13	0.0367 (18)	0.0402 (19)	0.0426 (19)	−0.0040 (15)	0.0094 (15)	0.0022 (15)
C14	0.0415 (19)	0.046 (2)	0.0420 (19)	−0.0066 (16)	0.0139 (16)	−0.0031 (15)
C15	0.056 (2)	0.063 (3)	0.051 (2)	−0.004 (2)	0.027 (2)	−0.0019 (19)
C16	0.064 (3)	0.071 (3)	0.045 (2)	−0.006 (2)	0.020 (2)	0.0082 (19)
C17	0.059 (3)	0.063 (3)	0.048 (2)	0.004 (2)	0.014 (2)	0.0117 (19)
C18	0.046 (2)	0.056 (2)	0.050 (2)	0.0023 (17)	0.0159 (18)	0.0049 (18)
C19	0.0339 (17)	0.0390 (18)	0.049 (2)	0.0023 (15)	0.0159 (16)	0.0002 (15)
C20	0.049 (2)	0.053 (2)	0.050 (2)	0.0000 (18)	0.0201 (19)	0.0005 (18)
C21	0.067 (3)	0.105 (4)	0.062 (3)	−0.029 (3)	0.024 (2)	−0.006 (3)
C22	0.060 (3)	0.063 (3)	0.077 (3)	0.002 (2)	0.043 (2)	0.008 (2)

### Geometric parameters (Å, °)

**Table:**

Br1—C4	1.906 (4)	C7—C8	1.441 (5)
Mn1—O2	1.851 (3)	C8—C9	1.357 (6)
Mn1—O1	1.945 (2)	C8—H8	0.9300
Mn1—N1	2.043 (3)	C9—C10	1.352 (6)
Mn1—N2	2.175 (3)	C9—H9	0.9300
Mn1—O3	2.190 (3)	C10—C11	1.387 (6)
Mn1—Cl1	2.3875 (11)	C10—H10	0.9300
O1—C7	1.308 (4)	C11—H11	0.9300
O2—C14	1.317 (4)	C12—H12	0.9300
O3—C20	1.227 (5)	C13—C19	1.371 (5)
N1—C12	1.315 (4)	C13—C18	1.412 (5)
N1—C1	1.419 (4)	C13—C14	1.447 (5)
N2—C19	1.302 (4)	C14—C15	1.352 (5)
N2—C2	1.369 (4)	C15—C16	1.372 (6)
N3—C1	1.294 (5)	C15—H15	0.9300
N3—C5	1.344 (5)	C16—C17	1.404 (6)
N4—C20	1.323 (5)	C16—H16	0.9300
N4—C21	1.414 (6)	C17—C18	1.322 (6)
N4—C22	1.431 (5)	C17—H17	0.9300
C1—C2	1.404 (5)	C18—H18	0.9300
C2—C3	1.404 (5)	C19—H19	0.9300
C3—C4	1.324 (5)	C20—H20	0.9300
C3—H3	0.9300	C21—H21A	0.9600
C4—C5	1.385 (5)	C21—H21B	0.9600
C5—H5	0.9300	C21—H21C	0.9600
C6—C7	1.362 (5)	C22—H22A	0.9600
C6—C11	1.416 (5)	C22—H22B	0.9600
C6—C12	1.438 (5)	C22—H22C	0.9600
			
O2—Mn1—O1	106.71 (11)	C9—C8—H8	118.8
O2—Mn1—N1	161.27 (12)	C7—C8—H8	118.8
O1—Mn1—N1	88.18 (11)	C10—C9—C8	120.2 (4)
O2—Mn1—N2	86.90 (11)	C10—C9—H9	119.9
O1—Mn1—N2	165.18 (11)	C8—C9—H9	119.9
N1—Mn1—N2	77.35 (11)	C9—C10—C11	118.3 (4)
O2—Mn1—O3	86.03 (11)	C9—C10—H10	120.8
O1—Mn1—O3	84.97 (11)	C11—C10—H10	120.8
N1—Mn1—O3	83.99 (11)	C10—C11—C6	123.5 (4)
N2—Mn1—O3	90.27 (11)	C10—C11—H11	118.3
O2—Mn1—Cl1	95.93 (9)	C6—C11—H11	118.3
O1—Mn1—Cl1	95.06 (9)	N1—C12—C6	127.6 (3)
N1—Mn1—Cl1	93.95 (9)	N1—C12—H12	116.2
N2—Mn1—Cl1	89.19 (8)	C6—C12—H12	116.2
O3—Mn1—Cl1	177.93 (8)	C19—C13—C18	115.9 (3)
C7—O1—Mn1	133.6 (2)	C19—C13—C14	123.2 (3)
C14—O2—Mn1	133.8 (2)	C18—C13—C14	120.8 (3)
C20—O3—Mn1	123.7 (3)	O2—C14—C15	118.8 (4)
C12—N1—C1	121.2 (3)	O2—C14—C13	124.6 (3)
C12—N1—Mn1	124.0 (2)	C15—C14—C13	116.5 (3)
C1—N1—Mn1	114.7 (2)	C14—C15—C16	120.8 (4)
C19—N2—C2	119.6 (3)	C14—C15—H15	119.6
C19—N2—Mn1	125.5 (2)	C16—C15—H15	119.6
C2—N2—Mn1	114.5 (2)	C15—C16—C17	123.1 (4)
C1—N3—C5	116.6 (3)	C15—C16—H16	118.4
C20—N4—C21	121.4 (4)	C17—C16—H16	118.4
C20—N4—C22	123.9 (4)	C18—C17—C16	118.0 (4)
C21—N4—C22	114.7 (4)	C18—C17—H17	121.0
N3—C1—C2	122.6 (3)	C16—C17—H17	121.0
N3—C1—N1	119.0 (3)	C17—C18—C13	120.7 (4)
C2—C1—N1	118.4 (3)	C17—C18—H18	119.7
N2—C2—C1	114.1 (3)	C13—C18—H18	119.7
N2—C2—C3	126.0 (3)	N2—C19—C13	125.1 (3)
C1—C2—C3	119.9 (3)	N2—C19—H19	117.4
C4—C3—C2	116.7 (3)	C13—C19—H19	117.4
C4—C3—H3	121.7	O3—C20—N4	126.5 (4)
C2—C3—H3	121.7	O3—C20—H20	116.7
C3—C4—C5	120.3 (3)	N4—C20—H20	116.7
C3—C4—Br1	118.9 (3)	N4—C21—H21A	109.5
C5—C4—Br1	120.9 (3)	N4—C21—H21B	109.5
N3—C5—C4	123.9 (4)	H21A—C21—H21B	109.5
N3—C5—H5	118.1	N4—C21—H21C	109.5
C4—C5—H5	118.1	H21A—C21—H21C	109.5
C7—C6—C11	117.6 (3)	H21B—C21—H21C	109.5
C7—C6—C12	123.7 (3)	N4—C22—H22A	109.5
C11—C6—C12	118.6 (4)	N4—C22—H22B	109.5
O1—C7—C6	122.2 (3)	H22A—C22—H22B	109.5
O1—C7—C8	119.9 (4)	N4—C22—H22C	109.5
C6—C7—C8	118.0 (3)	H22A—C22—H22C	109.5
C9—C8—C7	122.5 (4)	H22B—C22—H22C	109.5

### Hydrogen-bond geometry (Å, °)

**Table:**

*D*—H···*A*	*D*—H	H···*A*	*D*···*A*	*D*—H···*A*
C19—H19···Cl1^i^	0.93	2.81	3.691 (2)	159

Symmetry codes: (i) −*x*+1, −*y*+1, −*z*.
